# From seeds to trees: how E2 enzymes grow ubiquitin chains

**DOI:** 10.1042/BST20220880

**Published:** 2023-01-16

**Authors:** Adam J. Middleton, Catherine L. Day

**Affiliations:** Department of Biochemistry, School of Biomedical Sciences, University of Otago, Dunedin 9054, New Zealand

**Keywords:** E2 enzymes, protein degradation, ubiquitin, ubiquitin chains, ubiquitin signalling

## Abstract

Modification of proteins by ubiquitin is a highly regulated process that plays a critical role in eukaryotes, from the construction of signalling platforms to the control of cell division. Aberrations in ubiquitin transfer are associated with many diseases, including cancer and neurodegenerative disorders. The ubiquitin machinery generates a rich code on substrate proteins, spanning from single ubiquitin modifications to polyubiquitin chains with diverse linkage types. Central to this process are the E2 enzymes, which often determine the exact nature of the ubiquitin code. The focus of this mini-review is on the molecular details of how E2 enzymes can initiate and grow ubiquitin chains. In particular, recent developments and biochemical breakthroughs that help explain how the degradative E2 enzymes, Ube2s, Ube2k, and Ube2r, generate complex ubiquitin chains with exquisite specificity will be discussed.

## Introduction

**Figure 1.**
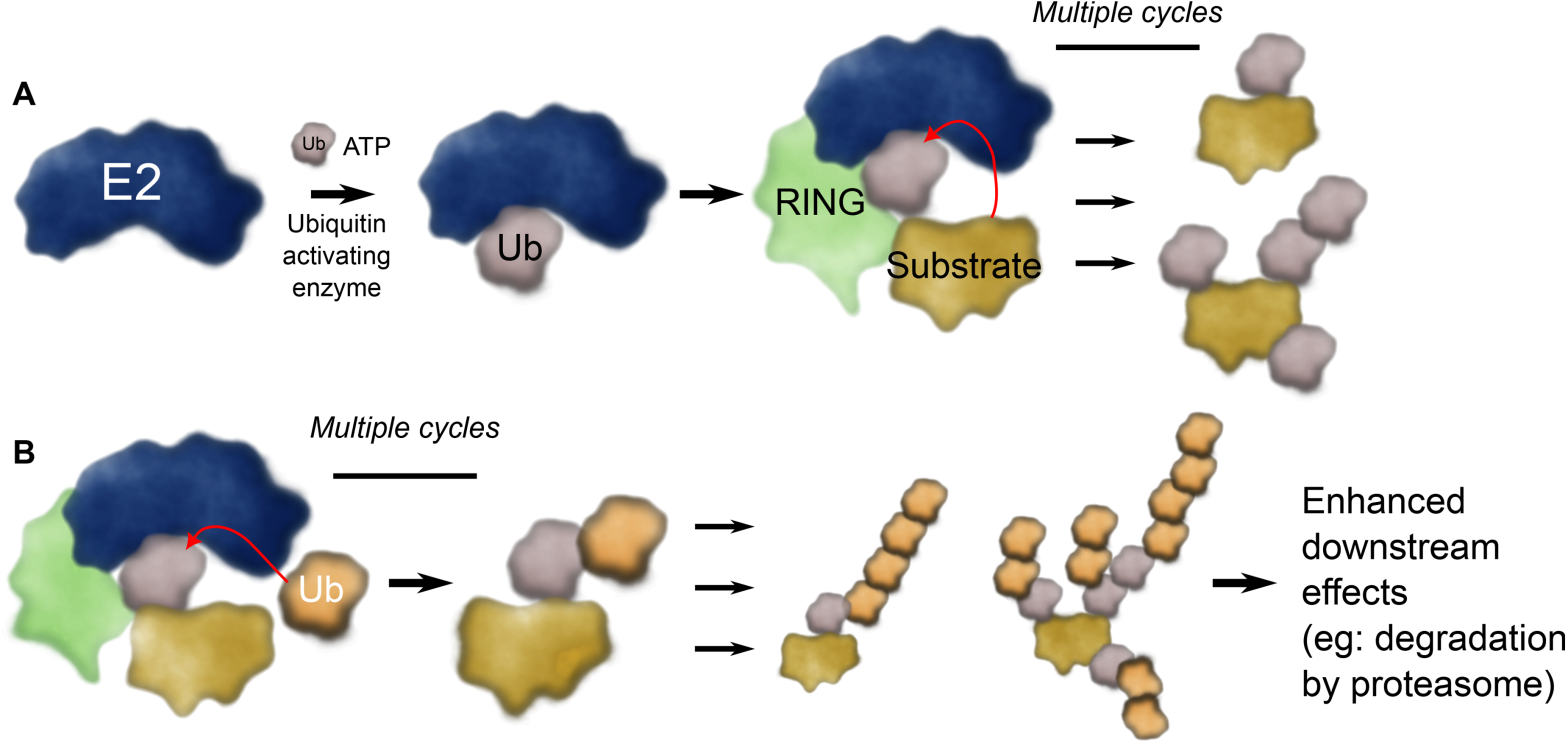
Overview of chain initiating and chain elongating E2 enzymes. (**A**) E2 enzymes are charged with ubiquitin by a ubiquitin activating E1 enzyme. The resulting E2∼Ub conjugate is usually activated by interacting with an E3 ligase (indicated as RING) that promotes ubiquitin transfer. For initiating E2 enzymes, they add a single ubiquitin molecule or short chains to substrate proteins. (**B**) Chain elongating E2 enzymes add particular ubiquitin chains to the initiating ubiquitin marks (i.e.: use ubiquitin as a substrate) and these signals enhance the downstream effect on the substrate protein.

The post-translational modification of proteins by ubiquitin is involved in most cellular processes. The ubiquitin system is most well-known for its role in managing protein degradation [1], however ubiquitin transfer (ubiquitylation) has extensive non-proteolytic roles including the recruitment of signalling complexes [2], and regulating the cellular localisation of proteins [3]. Proteins can be modified by single ubiquitin moieties or by polyubiquitin chains of eight different linkages [4]. Attachment of ubiquitin involves a cascade of three families of proteins. Humans have two E1, ∼40 E2, and >600 E3 ligase enzymes which sequentially activate, conjugate, and covalently link ubiquitin to a primary amine — typically a lysine (Lys) — in a protein substrate (Figure 1A). In parallel there are 10+ ubiquitin-like modifiers, which are also integral to cellular function [5]. Together the ubiquitin and ubiquitin-like cascade modifies an enormous range of substrate proteins and thereby directs their fate.

The ubiquitin code is diverse, and recent publications have highlighted how complicated and intertwined ubiquitin modification can be (e.g.: [6–12]). While Lys48-linked ubiquitin chains have long been known to result in degradation of the attached substrate protein by the proteasome [13], and this process is an essential part of recycling of misfolded proteins, maintenance of protein homeostasis, and control of the cell cycle [14], Lys11-linked ubiquitin chains are also strongly associated with degradation by the proteasome [15,16]. However, it is becoming clearer that branched (or heterotypic) ubiquitin chains form an integral part of the ubiquitin code [17] (Figure 1B). For example, branched chains were recently shown to result in efficient degradation of the cellular inhibitor of apoptosis 1 (cIAP1) protein, while the linear ubiquitin chain assembly complex (LUBAC) generates oxyester-linked branches off of linear ubiquitin chains [6,11]. Indeed, branched ubiquitin chains can amplify — or alter — the response of the cell to the ubiquitin signal [6,16–22].

## The central role of E2 enzymes

**Figure 2.**
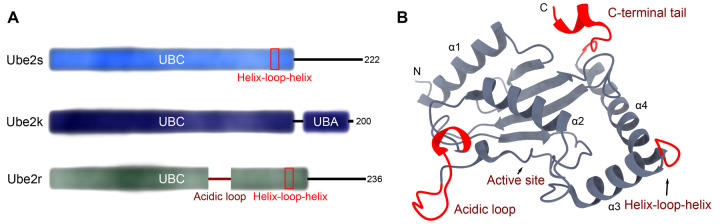
Structure of Ube2s, Ube2k, and Ube2r. (**A**) Domain structure of the E2 enzymes where flexible regions of the E2 enzymes are indicated with lines and domains are shown as coloured boxes. The numbers indicate the number of amino acids in the E2 enzymes. (**B**) Representative chain-building E2 enzyme (Ube2r; PDB 6NYO) showing the acidic insertion, C-terminal extension, and the helix-loop-helix motif in red. Alpha-helices are numbered, and the N and C terminus are indicated by N and C, respectively.

All E2 enzymes contain a catalytic domain called a ubiquitin conjugating (UBC) domain [23]. Many E2 enzymes contain only a UBC domain, while others contain additional N- or C-terminal extensions (or both) that play roles in regulating processivity or binding to other proteins like E3 ligases or substrate proteins (Figure 2). In the cell, E2 enzymes will normally be conjugated to ubiquitin where the catalytic Cys of the E2 enzyme is linked to the C terminus of ubiquitin by a thioester bond [24] (denoted E2∼Ub where ∼ indicates a thioester bond between ubiquitin and the active site Cys of an E2 enzyme). In the absence of an E3 ligase, the ubiquitin molecule in these E2∼Ub conjugates tends to occupy multiple catalytically inactive positions [25]. While some E2 enzymes can promote ubiquitin transfer in the absence of an E3 ligase, the reaction is dramatically accelerated by specific interactions with an E3 ligase. This is because when an E2∼Ub conjugate is bound by a Really Interesting New Gene (RING) E3 ligase, an interaction between ubiquitin and alpha-helix 2 of the E2 enzyme (Figure 2B) locks ubiquitin in a position so it is primed for catalysis [26–35]. This arrangement of the E2∼Ub conjugate is referred to as the ‘closed conformation’. For RING-catalysed ubiquitin transfer, the E2 enzyme typically determines the exact nature of the ubiquitin code.

## Ubiquitin as the substrate of ubiquitylation

For the formation of a substrate-attached ubiquitin chain, often a particular — and often promiscuous — E2 enzyme adds the initiating ubiquitin to the substrate protein (Figure 1A). Subsequently, another specific chain elongating E2 enzyme will build precise ubiquitin chains on these ubiquitin ‘seeds’ (Figure 1B). The act of generating a ubiquitin chain requires highly specific, but weak interactions between an incoming substrate (or acceptor) ubiquitin and an E2 enzyme. As expected for such highly efficient enzymes, the interaction between the acceptor ubiquitin and the E2 enzyme is weak and transient, with binding constants often >500 µM [36–39]. These weak interactions have hampered investigations into the precise mechanism of ubiquitin chain assembly. To circumvent this problem, multiple studies have used molecular modelling coupled with biochemistry to map the likely interaction of an incoming substrate ubiquitin molecule on the E2. Recently, however, major breakthroughs in cryogenic electron microscopy (cryoEM) and X-ray crystallography have helped these predictions considerably. In the subsequent sections, we highlight recent developments that have revealed how ubiquitin chains are formed by the chain-building E2 enzymes, Ube2s, Ube2k, and Ube2r (Figure 2).

## Lys11 chain formation by Ube2s and APC/C

The anaphase-promoting complex (APC/C) is a multi-subunit 1.2 MDa E3 ligase that is required for the transition from metaphase to anaphase [40–42]. The activity of the APC/C is highly regulated, and disruptions to its activity in humans can result in chromosome instability and cancer. When activated, the APC/C recruits the E2 enzyme, Ube2c, which initiates chain formation. Not only can Ube2c add priming ubiquitin molecules directly to substrates of APC/C, it will often add more than one ubiquitin to each substrate (multimonoubiquitylation), as well as short chains with varying topology [20]. In parallel with Ube2c, APC/C recruits Ube2s, which recognises the ubiquitin seeds planted by Ube2c and uses these to generate Lys11-linked ubiquitin chains. The activity of Ube2s results in densely decorated substrate proteins and this results in rapid and efficient degradation of substrates [20,43–45].

The APC/C complex contains a catalytic core including a RING E3 ligase domain, called APC11, and a cullin-like subunit, called APC2 [40]. While Ube2c binds to APC11 at the canonical RING-E2 interface, Ube2s makes distinct interactions with the APC/C. Notably, Ube2s is tethered to the APC/C via binding of a C-terminal extension of Ube2s to a groove between the APC2 and APC4 substrates of the APC/C [45–48]. Further contacts between the APC2 and the UBC domain of Ube2s precisely positions the E2 enzyme so it can catalyse transfer of ubiquitin to substrate-linked ubiquitin [46,49]. Recent work has demonstrated this unusual binding mode allows APC/C to engage both Ube2c and Ube2s simultaneously, which enables a feed-forward mechanism that ensures highly efficient ubiquitin transfer to substrates [50]. The unusual binding mechanism also allows APC/C to ‘track’ the tip, or distal end, of the Lys11 ubiquitin chain [51]. This provides an explanation for why the APC/C often appears to limit the length of the Lys11-linked ubiquitin chains to ∼six ubiquitin moieties [20,52].

Both NMR and X-ray data have been used to map the likely interface that ubiquitin uses against Ube2s when bound in the closed conformation [52,53]. Along with positioning of the donor ubiquitin, the formation of Lys11-linked chains by Ube2s is reliant on a precise interaction of the incoming acceptor ubiquitin and the active site of the E2 enzyme. Wickliffe et al. [52] showed that positioning of the acceptor ubiquitin relies predominantly on electrostatic interactions between the acceptor ubiquitin and the active site of Ube2s. Experiments demonstrated that while other Lys residues of ubiquitin could be positioned against the active site of Ube2s, these alternate arrangements were disfavoured by Ube2s. Subsequent evidence suggests that the APC11 RING subunit of the APC/C positions the incoming acceptor ubiquitin molecule adjacent to Ube2s [47,51]. It is likely that a combination of electrostatic contacts between Ube2s and ubiquitin, substrate-assisted catalysis, and external positioning of the acceptor ubiquitin by the APC/C allow Ube2s to exclusively produce Lys11-linked chains.

**Figure 3.**
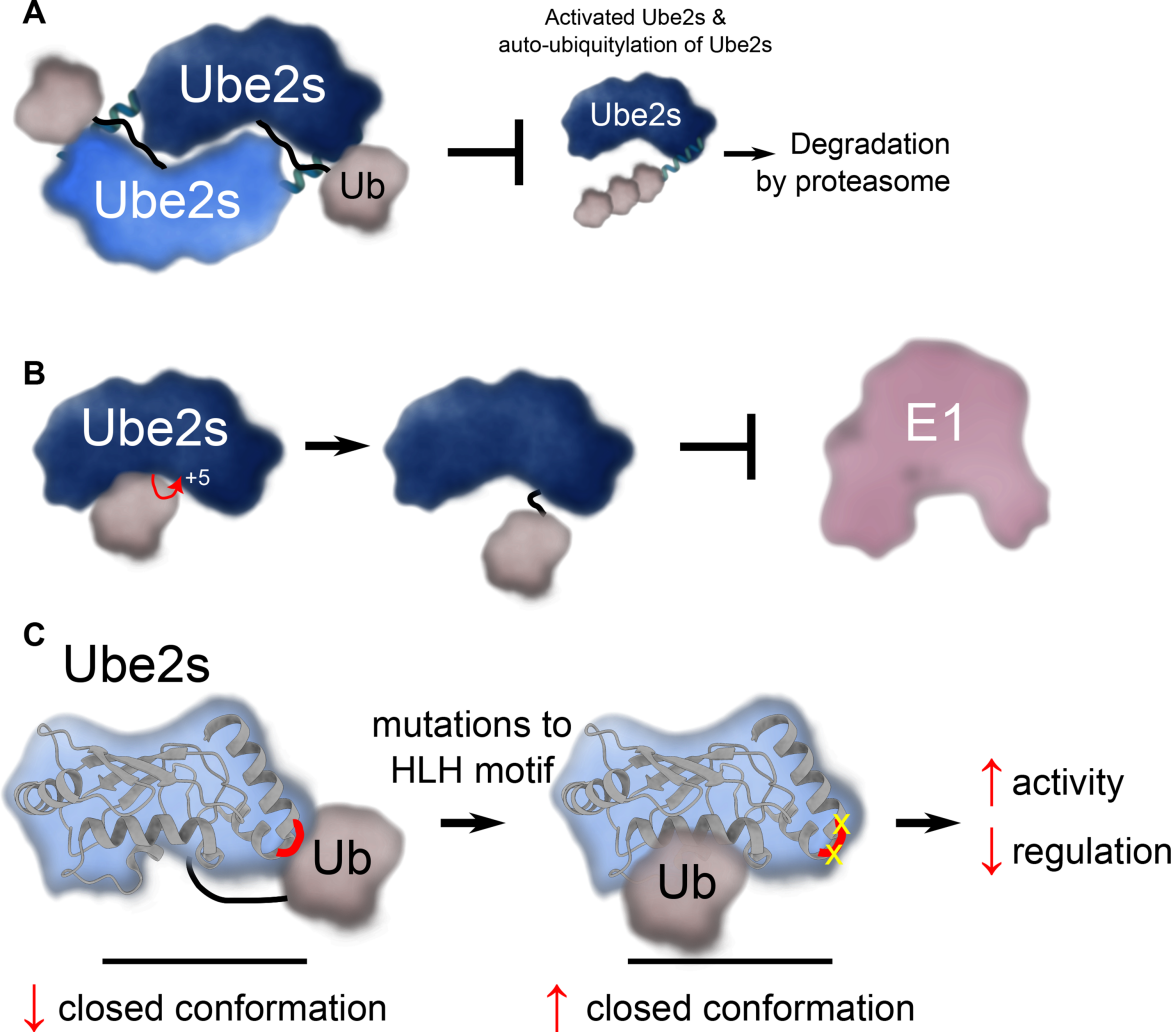
Regulation of Ube2s. (**A**) In the absence of recruitment by the APC/C E3 ligase, Ube2s can form a dimer that prevents the conjugated ubiquitin from binding Ube2s in the catalytically active closed conformation. The dimerisation blocks interaction with the E1 enzyme and Ube2s-catalysed ubiquitin transfer, including autoubiquitylation of Ube2s. (**B**) Ube2s can autoubiquitylate on a position five amino acids from its active site and thereby prevent Ube2s from being charged with ubiquitin by an E1 enzyme. (**C**) The helix-loop-helix region of Ube2s (indicated in red) has acidic residues that bind ubiquitin in a non-catalytic conformation to regulate its activity in the absence of the APC/C E3 ligase. Mutations (in yellow) that disrupt this site result in heightened activity, but decrease regulation.

Ube2s appears to autoregulate its activity in a number of ways. One mechanism of regulation involves the formation of a Ube2s–Ube2s dimer that blocks the ability of the conjugated ubiquitin from binding in the activated conformation (Figure 3A) [54]. Importantly, when Ube2s is in this dimeric configuration it appears resistant to proteolytic degradation, and may form a ‘reservoir’ that can be rapidly recruited when needed by the APC/C. In parallel, Ube2s can be inhibited by autoubiquitylation of a Lys residue adjacent to the active site (+5 Lys), a feature common to many E2 enzymes (Figure 3B) [55]. Autoubiquitylation at this site blocks interaction with the E1 enzyme and could be another way of reserving a pool of Ube2s that can be recruited when needed. What remains to be established is how the ubiquitin moiety on Ube2s is removed (presumably by a deubiquitinase) to allow charging by an E1 enzyme. Finally, Welsh et al. [39] recently investigated the role of acidic residues in the C-terminal helix-loop-helix motif in Ube2s. This structurally conserved region forms part of the interaction between Ube2s and APC2 in the APC/C complex [46,47,49]. Surprisingly, site-directed mutagenesis of the helix-loop-helix region results in heightened activity of Ube2s in the absence of the APC/C [39]. Molecular dynamics and NMR data revealed that the mutated Ube2s∼Ub conjugate is more likely to occupy the closed conformation. This surprising result suggests that residues in the helix-loop-helix of Ube2s actively prevent formation of the closed conformation by trapping an alternate conformation of the E2∼Ub conjugate [39]. It may be that Ube2s suppresses ubiquitin transfer in the absence of an E3 ligase to ensure it can only be active when bound to the APC/C (Figure 3C). Together, these features of Ube2s are likely to regulate the accessibility of Ube2s to the APC/C and ensure temporal control of the proper transition to anaphase.

## Ube2k: a Lys48-producing machine

The E2 enzyme, Ube2k (also known as HIP2 and E2-25K), is a chain-building E2 enzyme that produces only Lys48-linked ubiquitin chains [56]. C-terminal to its UBC domain Ube2k contains a ubiquitin associated (UBA) domain, which appears to be important for tethering Ube2k to substrates [10,57,58]. Changes in the activity and expression levels of Ube2k are associated with neurodegenerative disease and cancer [59–65]. Ube2k likely plays a broad role in cells by adding degradative Lys48-linked ubiquitin signals to various substrate proteins with a diverse selection of E3 ligases [32,66–68]. Recent work suggests Ube2k can also modify Lys63-linked ubiquitin chains to form branched chains, and this activity may be used to quench the Lys63-triggered signal [10,32].

Ube2k has a yeast homologue, Ubc1, which functions as a chain-extending E2 with the yeast APC/C in collaboration with a chain-initiating E2, Ubc4 (homologous to the Ube2d family of E2 enzymes) [69]. Like Ube2k, Ubc1 only produces Lys48-linked ubiquitin chains, and its activity with APC/C makes Ubc1 functionally equivalent to Ube2s. The first structure of a E2∼Ub conjugate in a closed conformation was determined for a Ubc1∼Ub conjugate in 2001 [70,71]. Furthermore, formation of Lys48-linked ubiquitin chains by Ubc1 was shown to be reliant on interactions between polar residues near its active site and the incoming substrate ubiquitin [72].

**Figure 4.**
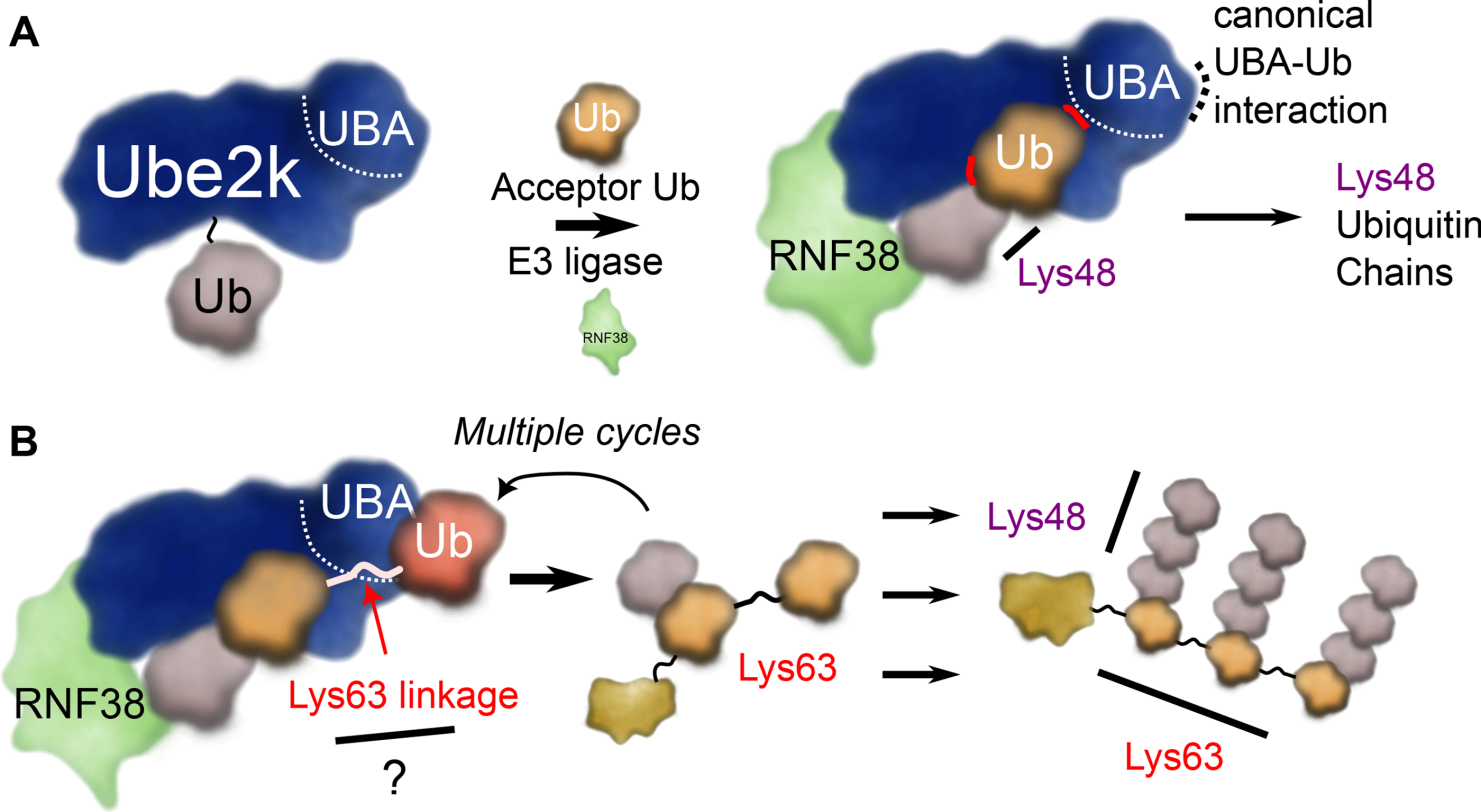
Ube2k generates Lys48-linked ubiquitin chains. (**A**) A second interface on the UBA domain of Ube2k helps position the acceptor ubiquitin (orange) on its active site (interactions highlighted in red) so it is oriented to produce Lys48-linked ubiquitin chains. (**B**) The UBA domain of Ube2k appears to interact with ubiquitin chains to promote formation of extensively branched chains.

Until recently, there was no structure of a Ube2k∼Ub conjugate in the closed conformation. While us and others have reported structures of the conjugate [68,73], the ubiquitin moiety was invariably bound to a neighbouring UBA domain in the crystal structure, similar to structures of Ube2k with free ubiquitin [74,75]. However, quite recently the Huang group reported a structure of a closed Ube2k∼Ub conjugate that was cross-linked with an acceptor ubiquitin at the active site of Ube2k [32]. This elegant study revealed that the acceptor ubiquitin is positioned by extensive interactions with a surface adjacent to the active site of Ube2k, as well as by an unexpected additional binding site on the UBA domain (Figure 4A). Critical interactions include polar contacts between the side chain of Arg54 from ubiquitin and main chain carbonyl oxygen atoms of residues 124 and 125 from Ube2k. Further contacts support the earlier docked model [68], and include hydrophobic contacts between Val87 and Thr88 from Ube2k and Thr55 from the acceptor ubiquitin. This study suggested the UBA domain could act to both anchor Ube2k to an established ubiquitin chain, while also positioning the acceptor ubiquitin adjacent to the active site (Figure 4B) [32]. Indeed, the authors suggest that wild-type Ube2k may preferentially add Lys48 linkages to Lys63-linked ubiquitin chains, in support of the work by Pluska et al. [10].

## Synthesis of Lys48-linked ubiquitin chains by Ube2r

The Skp1-cullin-F-box (SCF) E3 ubiquitin ligases are members of the modular Cullin-RING ligase family (CRL) of E3 ligases [76,77]. The CRL E3 ligases comprise a large cullin scaffold protein that binds both an adaptor protein and a RING E3 ligase. The adaptor proteins recruit interchangeable substrate recognition proteins and together these allow the CRL E3 ligases to ubiquitylate a staggering number of substrate proteins. The substrates of the CRL family are typically marked for degradation by the proteasome. The SCF E3 ligase binds a RING E3 ligase, RING-box protein 1 (Rbx1) (or Rbx2) [78], which recruits chain initiating and chain elongating E2 enzymes [79]. For initiation, the SCF E3 ligases typically use the promiscuous Ube2d family of E2 enzymes [80] (though recently it has been shown to also use a RING-between-RING (RBR) E3 ligase, ARIH1 [81,82]) before recruiting the Ube2r family of E2 enzymes to generate Lys48-linked ubiquitin chains.

**Figure 5.**
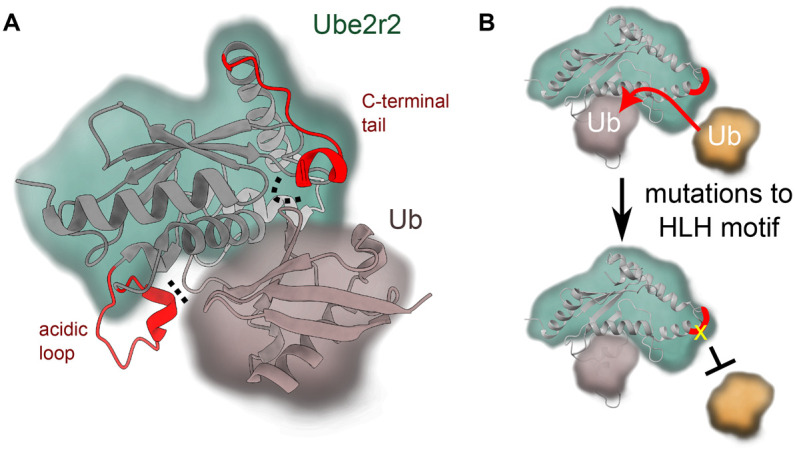
Ube2r2 positions the donor ubiquitin and regulates its activity. (**A**) The acidic loop and C-terminal domain (both in red ribbons, interactions indicated by dotted lines) of Ube2r2 can help position ubiquitin in the closed conformation (PDB 6NYO). (**B**) The helix-loop-helix motif (HLH, in red) on Ube2r2 appears to be important for positioning of the acceptor ubiquitin. Mutations (yellow) at this site result in a decrease in acceptor ubiquitin binding and activity.

The Ube2r family of E2 enzymes contains an acidic loop adjacent to the active site and an extended tail C-terminal to the UBC domain (Figure 2). The acidic loop has been demonstrated to be important for activity, and has been shown to form contacts with Rbx1 and the donor ubiquitin [83]. The molecular details that underlie the role of both the acidic loop and the C-terminal tail of Ube2r2 were recently revealed by a crystal structure of a Ube2r2∼Ub conjugate in the presence of a small molecule inhibitor, CC0651 [84,85]. In this structure, both the acidic loop of Ube2r2 and parts of its flexible C-terminal tail were visible in the density map, and these highlighted their dual roles in promoting ubiquitin transfer [85]. Notably, the resolved acidic loop makes an ionic contact with the donor ubiquitin between Glu112 in the flexible loop and Arg74 of ubiquitin, and this is likely important to help stabilise the C-terminal tail of ubiquitin (Figure 5A). Whereas the C-terminal tail reaches around the UBC of Ube2r2 to help bridge the UBC and the donor ubiquitin. A cluster of hydrophobic residues in the C-terminal tail contact the UBC of Ube2r2 and these position Tyr190 from Ube2r2 to act as a pin to bridge the UBC and the donor ubiquitin (Figure 5A). Together, these contacts help stabilise the E2∼Ub in the closed conformation. Indeed, disruption of the hydrophobic pocket or the Tyr190-UBC interaction inhibits activity, both in the presence and absence of an E3 ligase.

An earlier report suggested that binding of the acceptor ubiquitin to Ube2r is reliant on a flexible loop from ubiquitin [86]. Subsequently, charge-swap experiments demonstrated that the side chain of Arg54 from the incoming acceptor ubiquitin formed a polar contact with the side chain of Asp143 from Ube2r [83]. Furthermore, it was recently reported that Phe45, Ala46, and Gly47 from the acceptor ubiquitin, as well as Tyr87, His98, and Ser138 from Ube2r2 form essential contacts that help formation of Lys48-linked ubiquitin chains [85]. Mutations to the helix-loop-helix motif of Ube2r2 were also shown to reduce the ability of the acceptor ubiquitin to bind to the E2 enzyme (Figure 5B) [39]. While the helix-loop-helix is distant from the expected acceptor ubiquitin binding site, mutations to this site may disrupt the positioning of a ‘gate loop’ adjacent to the active site. This disruption may dampen binding of the acceptor ubiquitin. Regulating the dynamics of the gate loop — which have been shown to be important for activated E2 enzymes [38,87,88] — could be important for binding the acceptor ubiquitin.

## Summary and conclusions

The human genome encodes ∼40 E2 enzymes, of which all are characterised by having a highly conserved UBC domain. Some E2 enzymes only add ubiquitin to substrates, others exclusively produce ubiquitin chains, while some are promiscuous and can do both. While some of these differences can be explained by motifs in addition to the UBC, it is clear that small sequence differences between E2 enzymes also underpin functional plasticity. The intricate molecular contacts between E3 ligases and E2 enzymes suggests the balance of substrate chain initiation and chain elongation can be rapidly triggered and quenched, and how this process is regulated is critical for healthy cells. While a greater understanding of this process has recently emerged from structural biology techniques including cryoEM, X-ray crystallography, chemical biology, and biochemistry, there remains much to be determined about the molecular details of ubiquitin transfer by E2 enzymes.

## Perspectives

- The proper functioning of the ubiquitin system is essential for eukaryotic life. Disruptions to the ubiquitin system (including E2 enzymes) is of considerable interest to researchers due to their involvement with disease.
- For RING-catalysed ubiquitylation, the E2 enzymes determine the exact ‘language’ of the ubiquitin code. Some E2 enzymes plant the initiating ubiquitin on a substrate protein, while others build ubiquitin chains on these ‘seeds’ and these chains lead to a downstream effect like proteolytic degradation.
- While some E2 enzymes are well investigated, there still remain many whose roles and mechanism of action are still not clear. Comprehending how E2 enzymes produce ubiquitin chains with a high degree of specificity on diverse substrates, and how they are regulated are fundamental questions that continue to be investigated.

## Abbreviations

APC: anaphase-promoting complex
CRL: Cullin-RING ligase family
RING: Really Interesting New Gene
SCF: Skp1-cullin-F-box
UBA: ubiquitin associated
UBC: ubiquitin conjugating
